# Short-term responses of small mammal diversity to varying stand-scale patterns of retention tree patches

**DOI:** 10.1371/journal.pone.0273630

**Published:** 2022-08-31

**Authors:** Sean M. Sultaire, Andrew J. Kroll, Jake Verschuyl, Gary J. Roloff

**Affiliations:** 1 Department of Fisheries and Wildlife, Michigan State University, East Lansing, MI, United States of America; 2 Weyerhaeuser, Springfield, OR, United States of America; 3 National Council for Air and Stream Improvement, Anacortes, WA, United States of America; Southeastern Louisiana University, UNITED STATES

## Abstract

Retention forestry is a common practice for biodiversity conservation in forests managed for wood production. Retention forestry often leaves unharvested patches of trees that vary in size and spatial pattern but experiments evaluating the effects of different retention patch configurations at a constant level of retention are lacking for many regions and taxonomic groups. We implemented an experimental study in clearcut conifer stands with retention across the U.S. Pacific Northwest region. The study consisted of five stand-level (11–55 ha) experimental treatments each replicated 10 times within a randomized complete block design, resulting in 50 treated stands. Retained tree density was comparable across treatments but size, number, and location (upland or riparian) of patches within stands varied among the five treatments. Within experimental treatments, we measured small mammal (<1kg) species and functional trait (i.e., body size, diet, activity stratum) richness in retention patches, surrounding harvested portions of stands, and nearby unharvested stands. We evaluated species and functional trait richness by treatment using generalized linear mixed-effects models and species-specific responses to retention placement using a community occupancy model. We obtained repeat captures of 21 species of small mammals but found limited evidence of a treatment effect on species richness, and no differences in functional trait richness. Species richness was highest where all retained trees were aggregated into one patch placed adjacent to a forested riparian buffer (mean = 6.6 species, 95% CI = 5.7–7.5), and lowest in the treatment containing one retention patch in the upland portion of a harvested stand (mean = 4.7 species, 95% CI = 3.8–5.6). Furthermore, estimates of species richness within retention patches of harvested stands (i.e., not considering species in harvested areas) did not differ among treatments, indicating that the slightly elevated species richness in riparian-associated retention results from 1–2 species in these patches that do not occur in adjacent harvested portions of each treated stand. Patch occupancy of several species was higher in riparian patches than harvested portions of the treated stands, and fewer species had higher occupancy in upland patches compared to harvested portions of treated stands. Our results indicated that at retention densities currently required in Oregon and Washington, the location of retention patches had a small influence on stand-scale measures of small mammal diversity, but local increases in species richness may be obtained by retaining trees adjacent to riparian buffers.

## Introduction

Society is increasingly reliant on intensively managed, even-aged rotations of forest plantations to meet demand for wood and other forest products [1, 2]. Consequently, the area of these plantation forests is increasing despite decreases in global forest cover [1], creating opportunities for forest species conservation. However, wildlife habitat structure in tree plantations is simplified compared to less intensively managed forests [3], and early seral conditions following forest harvest are less diverse compared to forests resulting from natural, stand-replacing disturbances [4]. Retention forestry aims to increase biodiversity value of plantation forests by leaving behind a proportion of trees and downed and standing dead wood (i.e., snags) during forest harvesting [5], as dead wood is a critical resource for many forests species that is lacking in managed forests. Retention forestry typically increases species richness of wildlife communities within harvested forests compared to older forest interiors and harvested forests without retention [6–9]. However, in the context of harvested stands containing aggregated retention, where retained trees and snags are grouped in distinct patches, effects of retention patch size and location on wildlife communities are less clear [8].

Whether conserving a single-large or several-small (SLOSS) patches of habitat conserves more species at similar amounts of habitat has been debated for decades [10–12]. However, effects of patch size and number at a constant level of retention have received little attention in experimental retention forestry studies. Species richness patterns are often driven by environmental heterogeneity [13] and grouping trees in several small patches may increase environmental heterogeneity by encompassing different microhabitats across the harvested stand. Alternatively, aggregating retention trees into a single large patch may maintain conditions necessary for forest interior species, whose persistence following forest harvest would influence species richness at the scale of the entire harvested area [14, 15]. Available evidence suggests that retention patch size and number do not influence species richness and abundance within harvested forests [16, 17]. However, these studies were limited in geographic scope and potential effects of retention patch size and number on biodiversity have not been evaluated in many important timber producing regions (e.g., North American conifer forests). Thus, furthering the taxonomic and geographic breadth for effects of different aggregated retention strategies can support informed decisions regarding retention placement to meet biodiversity conservation objectives in managed forests.

Measuring species-specific responses to retention practices provides inference regarding the contribution of species-level responses to community level patterns and which species gain or lose from a given retention strategy. Furthermore, species traits can influence their response to forest patch size [18]. Hence, functional richness, or the diversity of traits species in a community possess, can complement species richness when quantifying the effect of patch size and fragmentation on a community [19, 20]. Stronger or consistent responses of functional richness when compared with species richness to variation in patch size suggests that changes in patch size disproportionately affects certain functional groups [21]. This pattern is important because traits that species possess within a community are more strongly related to a community’s contribution to ecosystem function than species richness [22, 23]. In contrast, greater changes in species richness compared to functional richness suggest that variation in patch size only reduces levels of functional redundancy within a community [24].

**Fig 1:**
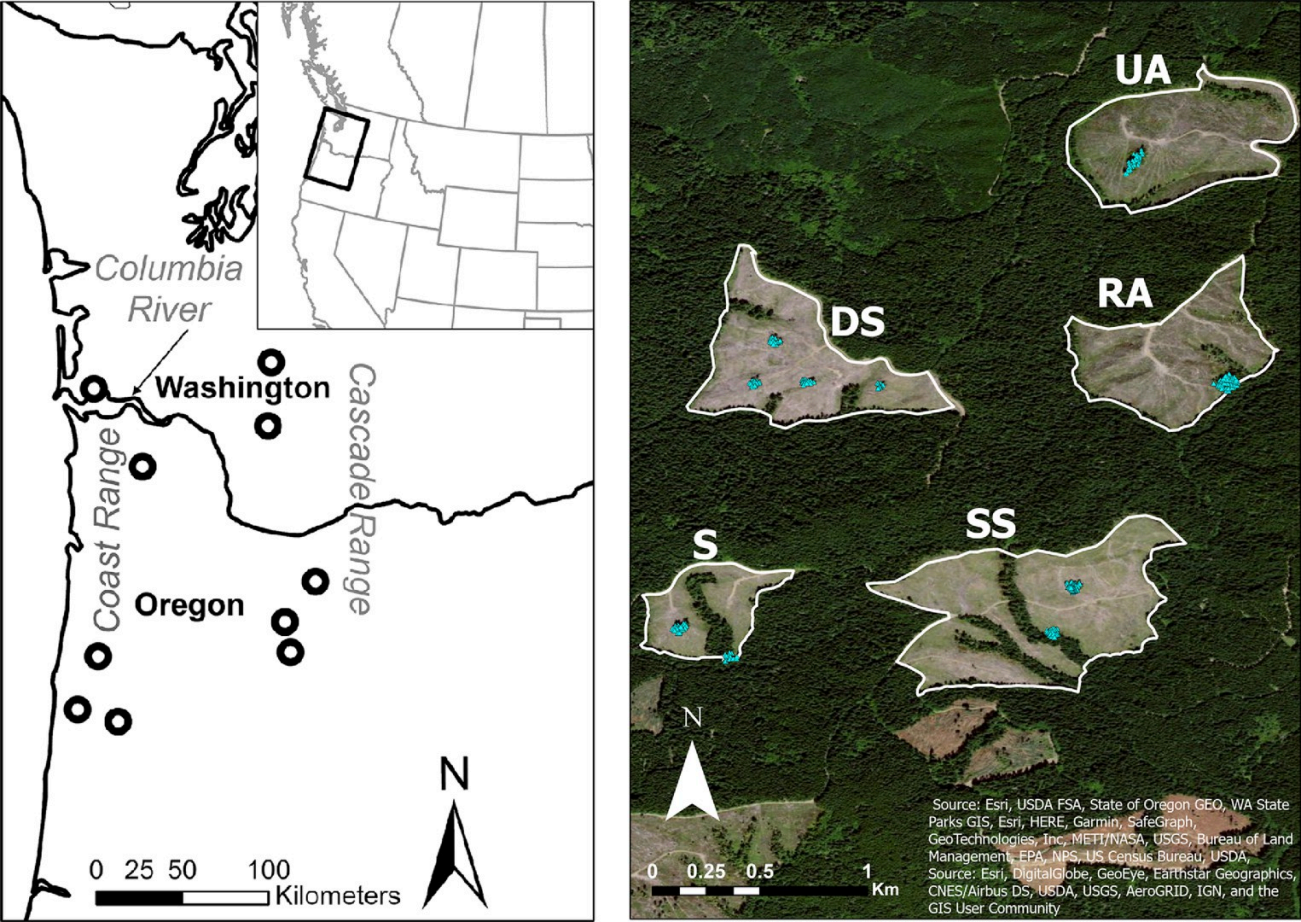
Location of ten experimental blocks with five structural retention treatments in each block (left panel) and aerial view of one experimental block showing each of the five experimental retention stands with the location of retained structures in each stand depicted by blue triangles (right panel), Oregon and Washington, U.S.A. Half of the retained structures in the SS and DS treatments consisted of created snags, shown in Fig 2. RA = Riparian Aggregated, UA = Upland Aggregated. S = Split; SS = Split with Snags; and DS = Dispersed with Snags.

We implemented an experimental study to test how the size, number, and location of retention patches influences species and functional richness of non-volant small mammals (<1 kg) within clearcut conifer forests with retention in the Pacific Northwest, U.S.A. The study included five retention treatments, each containing different patch sizes and combinations of upland retention and/or retention connected to forested riparian buffers (Fig 1). Treated stands contained a comparable density of tree retention relative to total harvested area, but the size, number, and location of retention patches differed, creating an opportunity for inference on small mammal community responses to patch fragmentation (i.e., SLOSS) in addition to stand scale retention forestry effects. Two of the five treatments also contained created snags, consisting of the lower ~10m of trees with crowns harvested, within patches. We predicted that harvested stands containing aggregated retention would provide habitat for forest interior species, in addition to species and functional groups present in harvested portions of the stand, thereby resulting in small mammal communities with high species and functional richness at the scale of the entire treated stand. We further predicted that the positive effect of aggregating retention would be strongest in the treatment containing aggregated riparian-associated retention due to connectivity with forested riparian buffers. Alternatively, small mammal species richness in harvested stands may be higher in several small patches if multiple patches encompass higher habitat diversity or allow competing species to coexist [25]. However, under this pattern, functional richness would be similar across treatments because competing species are functionally similar [26]. In addition to providing information on effects of forest harvesting practices on small mammal diversity in the Pacific Northwest, our results provide inference on the effects of forest patch pattern and connectivity on forest small mammal communities.

## Methods

### Study design

**Fig 2:**
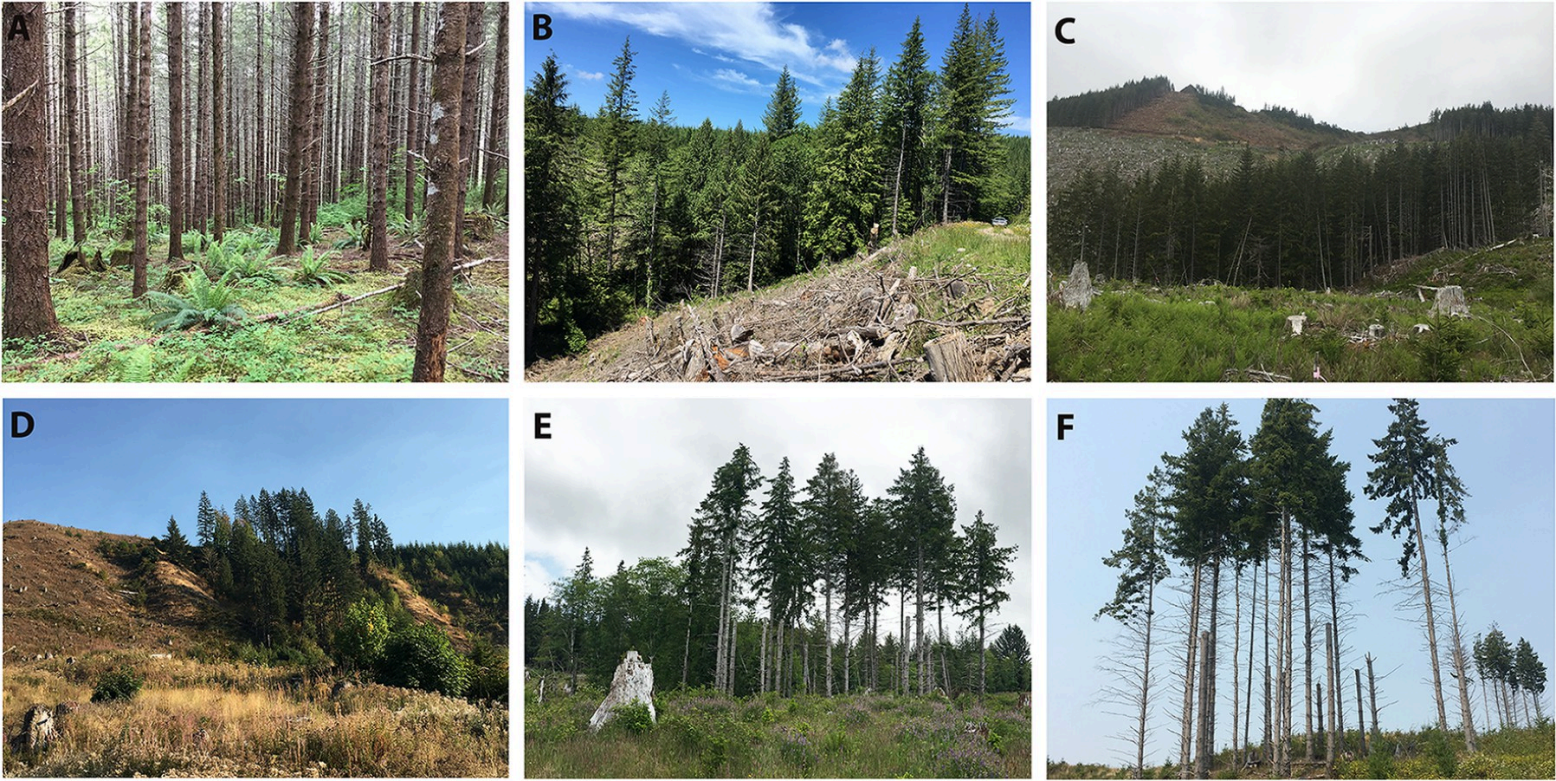
Examples of retention patches within clearcuts from the five experimental treatments and an unharvested rotation-aged stand (a). (b) a riparian-aggregated patch with riparian buffer zone in lower left, (c) an upland-aggregated patch in the center of a clearcut, (d) an upland patch from a split retention treatment adjacent to a regenerating stand, (e) a riparian patch from a split retention treatment with created snags and riparian buffer zone in lower left, and (F) a patch from the dispersed with snags treatment with a second patch within stand visible in background, northwest Oregon and southwest Washington, USA, 2017–2019.

We conducted this study in the mesic conifer forests of northwest Oregon and southwest Washington, U.S.A., in the Coastal and Cascade Mountain Ranges (Fig 1). Forest practices rules in both states set maximum clearcut harvest size to ~55 ha, require unharvested forested stream buffers, and require retention of ~5 green trees or snags/ha of the harvested area [27, 28]. Oregon does not provide rules on retention tree placement [28], whereas Washington requires that all locations within a clearcut harvest area are <244m from retention [27]. Within retention forestry systems, two distinct strategies of retention forestry are practiced, dispersed retention where trees are retained individually throughout the clearcut area, and aggregated retention where trees are retained grouped together in patches [8]. Our study focused on aggregated retention and different patch configurations with this retention system. In the context of this experiment, we refer to a *stand* as a continuous clearcut area (~11–55 ha) with retention and a *patch* as a tree group of variable size embedded in the stand. Our experimental design included 5 retention treatments that manipulated patch size and location within stands, as well as the presence of created snags (Fig 2), with complete replication in 10 blocks (Fig 1). The treatments included:

1. **Riparian Aggregated Treatment (RA):** All retention trees grouped together in one patch connected to an unharvested riparian protection zone (Fig 2B)
2. **Upland Aggregated Treatment (UA):** All retention trees grouped together in an upslope portion of the clearcut stand, sometimes on the edge adjacent to <15-year-old forest (Fig 2C)
3. **Split Treatment (S):** Half of the retained trees grouped in a patch connected to an unharvested riparian zone and half grouped in an upslope portion of the clearcut stand (Fig 2D)
4. **Split with Snags Treatment (SS):** Identical to the Split treatment but half of the retained trees were mechanically topped and turned into snags (Fig 2E)
5. **Dispersed with Snags Treatment (DS):** Retention trees grouped into at least 4 small patches (at least 15 trees) dispersed throughout the clearcut stand, with snags mechanically created within these patches (Fig 2F)

Aside from these treatment criteria, all other operational restrictions applied, including protection of waterbodies, threatened or endangered species locations, and special ecological or cultural sites, avoidance of unstable slopes, and soil conservation [27, 28]. Hence, our experimental design reflects typical site- and landowner-specific variability in clearcut harvesting practices in the region and makes the results applicable to managed forests across the region. At the commencement of sampling in 2017, time since harvest for each stand varied from 2 to 5 years. Consistent with typical forest regeneration practices in the PNW, following harvest each treatment stand received an herbicide application to control competing vegetation followed by planting of desirable native conifer seedlings, primarily Douglas fir (*Pseudotsuga menziesii*). Western hemlock (*Tsuga heterophylla*) was more common in mesic stands and occasionally planted, as was noble fir (*Abies procera*) in higher elevation stands (>900 m). A minor deciduous component of bigleaf maple (*Acer macrophyllu*m) and red alder (*Alnus rubrum*) was also present in certain stands.

**Fig 3:**
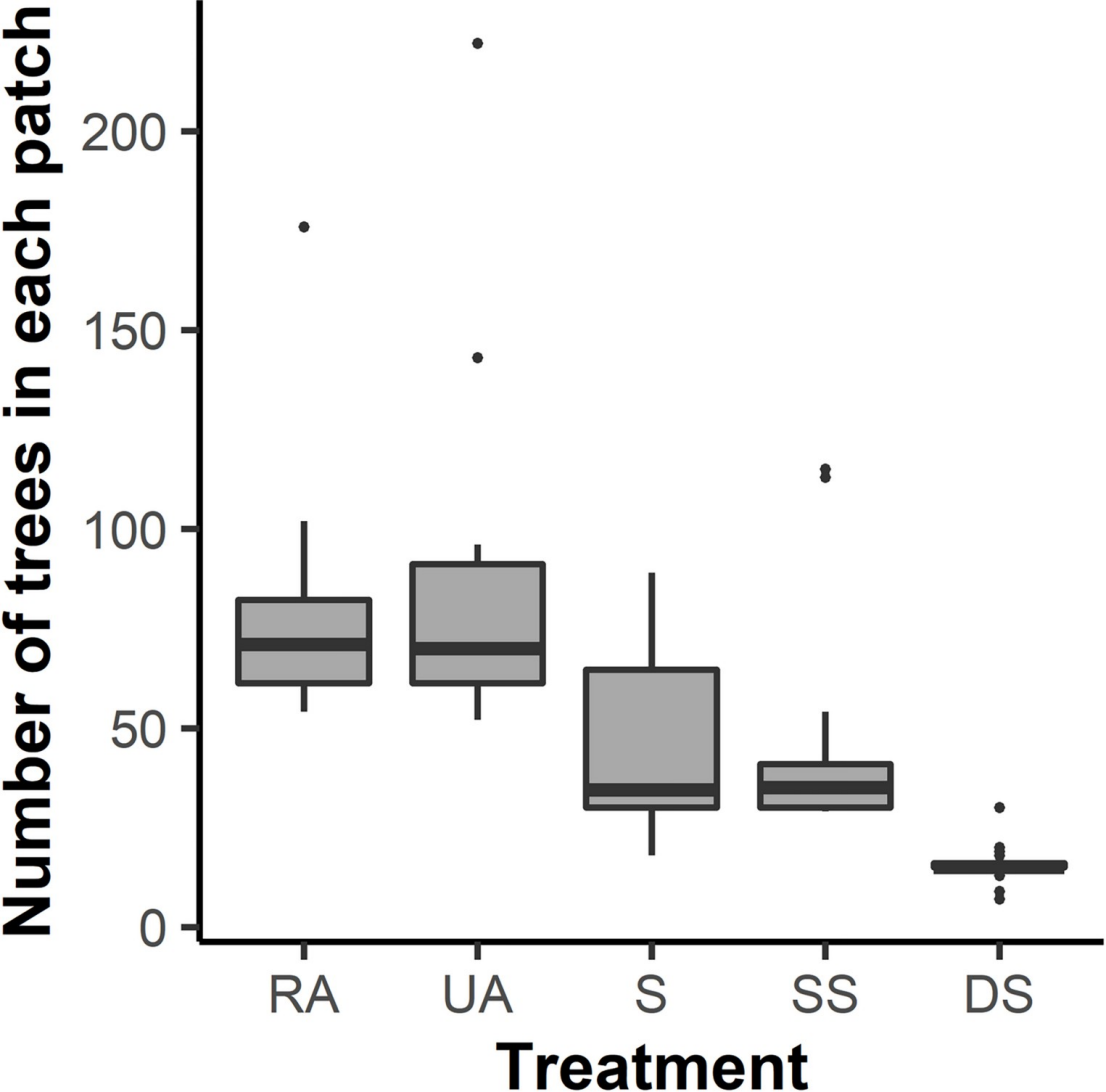
Box and whisker plots depicting variation in patch size among the five retention treatments, northwest Oregon and southwest Washington, USA, 2017–2019. In the box plots horizontal lines indicate the median, boxes represent interquartile range (IQR), and vertical lines represent 1.5 * IQR of patch sizes from each treatment type. Black points are outliers. Treatments were fully replicated in each of 10 experimental blocks (*n* = 50). Abbreviations are RA, Riparian Aggregated; UA, Upland Aggregated; S, Split; SS, Split with Snags; and DS, Dispersed with Snags. Stands with the S, SS, and the DS treatments contained multiple patches per stand; each RA and UA treatment stands only contained a single patch.

Mean distance between stands within an experimental block was 5.9 km compared to a mean distance of 125 km between stands among the different blocks. The size of treated stands varied from 11.4 to 55 ha (mean = 33.6, SD = 10.6 ha) and number of retained structures (i.e., green trees and created snags) per stand across all treatments ranged from 51 to 227 (mean = 82, SD = 35). Despite variation in patch size within treatments due to differences in the size of treated stands, large differences in patch size were apparent among treatments, particularly between the two aggregated and the dispersed treatment (Fig 3). In addition to sampling treatment stands, we sampled one rotation-aged forest (~50 years old) in nine of 10 blocks to compare small mammal communities occurring in managed, unharvested forests to those found in treatment areas (Fig 2A).

### Field methods

Within treatment stands, we paired live-trap grids of consistent size (48 trap sites) in retention patches and in adjacent harvested portion of stands (hereafter called clearcut), for a total of 96 trap sites per stand with 5m spacing between traps. In RA and UA treatments, we set two grids with 48 traps each: one within the retention and one within the clearcut. In S and SS, we set four grids of 24 traps: one in each patch and one in the clearcut in the vicinity of each patch. For DS, we set eight, 12-trap grids: four in retention patches and four in clearcut areas proximate to each patch (S1 Fig). Variation in retention patch sizes among the same treatment types resulted in minor variations in grid sizes, and considerable variation in grid configuration due to retention patch shape. We established trapping grids in clearcuts by following a random compass bearing and distance from the center of a sampled retention patch (S1 Fig). These distances varied from 12 to 213 m (average = 49.50, SD = 27.50). We sampled rotation-aged, unharvested stands with a 48-trap grid, consistent with effort in retention patches of experimental treatment stands.

We live-trapped each treatment for three consecutive summers (2017–2019) between late May and early September using a combination of Sherman (Model LFA, 7.6 x 8.9 x 22.9 cm; H. B. Sherman Traps, Inc., Tallahassee, Florida) and Tomahawk (Model 202, 48.3 x 15.2 x 15.2 cm, Tomahawk Live Trap Co., Tomahawk, Wisconsin) traps. Stands were only sampled once each year and to account for phenological changes in small mammal communities we rotated the date of sampling for each stand among the three years. Furthermore, we sampled stands from the same block within two weeks of one another each year, to remove consistent bias in the timing of sampling each treatment that could affect estimated treatment effects. Due to logistical constraints, we were unable to trap one UA treatment in 2019, and one Split treatment was sampled for only three nights in 2019. We trapped seven of nine rotation-aged stands in 2019 because two of the areas were logged between 2018 and 2019 as part of forest harvesting schedules. We placed Sherman traps at every trap site (5m spacing), whereas Tomahawk traps were placed at every-other trap site (10 m spacing), with trap locations consistent across years. We baited Sherman traps with a combination of black oil sunflower seeds, whole oats, and fresh apple. For Tomahawk traps, we baited with peanut butter, rolled oats, and apple. We also placed polyester batting in each trap to help captured animals thermoregulate.

We opened traps in the morning or afternoon and then checked the following morning or early afternoon for four consecutive days. During each trap check we identified captured animals to species, recorded weight, and tagged each ear with unique ear tags (National Band and Tag Company, Style 1005–1, Monel). For recaptures, we only recorded tag numbers and location of capture. For shrews, we collected morphological measurements to aid in species identification but did not tag them; we considered all captured shrews to be new individuals. As we did not bait traps to attract shrews, and >60% of shrews we captured died, we think this assumption is reasonable. We collected dead shrews for identification in the lab with the exception of the most common and easily identified species, Trowbridge’s shrew, (*Sorex trowbridgii)*. Shrews with a tail length < 42 mm were classified as vagrant shrew (*Sorex vagrans*), a species more characteristic of open habitats compared to the other shrew species in the region [29]. Trowbridge’s and vagrant shrews comprised 68% of total shrew captures. We classified all other shrews as *Sorex* species. These included individuals of Baird’s shrew (*Sorex bairdii*), montane shrew (*Sorex monticolus*), Pacific shrew (*Sorex pacificus*), and fog shrew (*Sorex sonomae*) that cannot be differentiated in the field and have largely allopatric distributions in western Oregon [29]. We differentiated between the two species of deer mice (*Peromyscus* spp) in the three Washington blocks by measuring tail length. Individuals with tail lengths ≥95mm were classified as Keene’s mouse (*Peromyscus keeni*), and those <95mm were classified as American deer mouse (*Permomyscus maniculatus*), which separates adults of each species with 94% accuracy [30]. Animal capture, handling and tagging procedures were approved by the Institutional Animal Care and Use Committee at Michigan State University (AUF 04-16-040). All necessary research permits were acquired from landowners, for field sampling and collection permits were obtains from Oregon Department of Fish and Wildlife and the Washington Department of Fish and Wildlife.

To explore how habitat heterogeneity differed across retention treatments, we used data collected on ground vegetation within treatment stands. During the summer of 2018, botany field crews surveyed ground vegetation in each treatment between May and September, in retention patches and adjacent clearcuts collocated with small mammal grids. Within 1m^2^ plots spaced every 7 m along 21–48 m long transects oriented in cardinal directions from a center location, crews classified the area of each herbaceous species into seven cover classes with midpoints of 0.5, 3, 15, 37.5, 62.5, 85, 97.5 percent. We grouped all fern, forb, and grass species each into separate functional groups, for a total of three herbaceous functional groups. Proportion of shrub cover on each transect was quantified as the proportion of transect length that was intercepted by shrubs >1m tall.

### Data analysis

We considered mammal species captured >1 time, except for non-native Virginia opossum (*Didelphis didelphis*; two captures) in the richness analyses. We excluded four species captured once because our trapping protocol did not reliably detect these widespread species (*Mephitis mephitis*, *Sylvalagus bachmanii*, *Aplodontia rufa*, *Microtus richardsoni*). Observed species richness of a location is usually lower than true richness and to account for potential under-sampling we used the Chao 1 species richness estimator [31, 32]. The Chao1 estimator adjusts observed species richness at a location upwards based on number of species represented by 1 or 2 individuals, under the assumption that rare species contain most of the information about the number of undetected species. We estimated species richness at two scales: treatment stand (i.e., stand-scale) and for retention patch and clearcut separately within a treatment stand (i.e., cover type-scale). Comparing estimates at these scales evaluates if redundancy between retention patch and clearcut communities exists and varies by treatment. For example, if species richness estimates for stand and cover type extents are similar, we can infer that most species are redundant between retention patches and clearcuts. We calculated the Chao1 estimator in R package *vegan* [33].

We estimated functional richness using a dendrogram constructed from mean body mass (grams), diet guild, and activity stratum (arboreal, semi arboreal, ground dwelling, or fossorial) using Gower’s distance and the unweighted pair group method with arithmetic mean clustering algorithm (S3 Fig) [34]. We calculated mean body mass for all individuals captured of a particular species, including juveniles. For species that we did not weigh in the field (i.e., carnivores), we sourced body mass from a regional mammal guide [35]. We grouped species into diet guilds based on whether they are primarily carnivorous (3 species), insectivorous (4 species), or granivorous/mycophagous (14 species). This functional trait was also phylogenetic at the order level as we grouped carnivores, insectivores, and rodents. Although we do not assert that these 3 traits entirely represent functional niches of these species in forest ecosystems, body size determines a large part of a species ecology within guilds [36]. Using the dendrogram constructed from these three traits, we quantified functional richness for treatment areas as the total branch length of a tree linking all species captured in a treatment stand during a given sampling session [37]. Although a Chao1 correction is available for functional richness, we only modeled observed functional richness because of the highly variable estimates of functional richness produced by this method. We performed functional richness calculations in R package *BAT* [38].

We used species and functional richness estimates for each treatment area as response variables in linear mixed effects models to quantify how retention pattern influenced mammalian diversity in and adjacent to retention patches. We used models with a Gaussian response for both stand-scale species and functional richness because both variables were approximately normally distributed. Species richness estimates for cover types within stands were left-skewed so we fit this variable as a Poisson distributed response, after rounding the Chao1 estimates to the nearest integer.

We structured our models using Helmert contrasts with the RA Treatment estimated as the intercept, and coefficients for remaining treatments representing deviations in richness from that treatment. We also included sampling year as a numeric covariate to account for potential changes in small mammal richness throughout the study time period. We fit a second model for each response variable that included an interaction between retention treatment and sampling year to test whether the effect of retention treatment on species and functional richness changed over the course of sampling. To account for seasonal changes in the small mammal community, we included week of sampling as a numeric covariate. Our data included sampling from the same sites across multiple years, so for each model we included a stand-level random effect to account for dependency among observations from the same stand in addition to a block level random effect. The treatment by year interaction model did not converge with a block-level random effect so we did not include variation at this level in the model. We related the four vegetation variables–shrub, forb, grass, and fern cover–to retention treatment and cover type (patch or clearcut) using a Gaussian mixed-effects models with a block level random effect. We used R package *lme4* to fit mixed effects models [38]. We assessed regression assumptions of all mixed-effects models using residual plots from the R package *DHARMa* [39].

### Species-specific responses

To explore how species-specific occurrence patterns contributed to stand-scale richness patterns in response to retention treatment, we developed a community occupancy model [40]. Occupancy models are hierarchical, logistic regression models that use repeat surveys of sites to estimate species occupancy and detection probabilities, conditional on occupancy, in relation to environmental and survey-specific covariates [41]. Community occupancy models estimate occurrence and detection for multiple species by assuming species-specific parameters follow a common distribution defined by a mean and standard deviation [40]. Using this approach, estimation of occupancy relationships even for rare species in a community with few detections is possible. For species detected infrequently, estimated occupancy will be similar to the community mean. An important assumption of occupancy models is that unmodeled heterogeneity in species detection probabilities does not exist and all relevant factors associated with detection are included in the model [41]. As differences in local species abundance can influence detection probability, we chose a model formulation that allowed for abundance-induced heterogeneity in species detection probability [42, 43].

We fit the community occupancy model to species detection data at trapping grids within stands, a total of 604 subplots. We included grid location as a categorical covariate on occupancy, indicating whether the grid occurred in clearcut, upland retention, riparian retention, or rotation-age forest. In addition to allowing abundance-induced detection heterogeneity, we also fit sampling week as a numeric detection covariate. Stand was included as a random intercept on occupancy to account for repeated measures across years. To assess consistency between our models, we used posterior predictive checks and did not find any evidence for lack of model fit (S1 Appendix). The priors we used in the analysis are included with the R code for the model in S1 Appendix.

## Results

We captured 5,150 individual mammals of 24 species and, as typical for small mammal studies, the distribution of abundances skewed heavily towards a few common species (S2 Fig). Four species accounted for over 85% of all captures: *Peromyscus maniculatus* (2,065 individuals), *Neotamias townsendii* (1,434 individuals), *Microtus oregoni* (656 individuals), and *Sorex trowbridgii* (252 individuals). Number of individual animals captured over four-day trapping sessions ranged from 3 to 123 (median = 29). Observed species richness at the stand-scale ranged from 1 to 9 species (median = 5), Chao1 estimates ranged from 1 to 14 species (median = 5) and stand scale functional richness (dendrogram length) ranged from 0.62 to 2.09 (median = 1.25). Observed cover type-scale mammal species richness ranged from 1 to 8 species (median = 4), and Chao1 estimates for this scale ranged from 1 to 13 species (median = 4). Estimated random effects did not indicate any strong spatial structuring of small mammal species or functional richness at either the block or stand scale, although one block did have significantly lower functional richness (S4 Fig).

**Fig 4:**
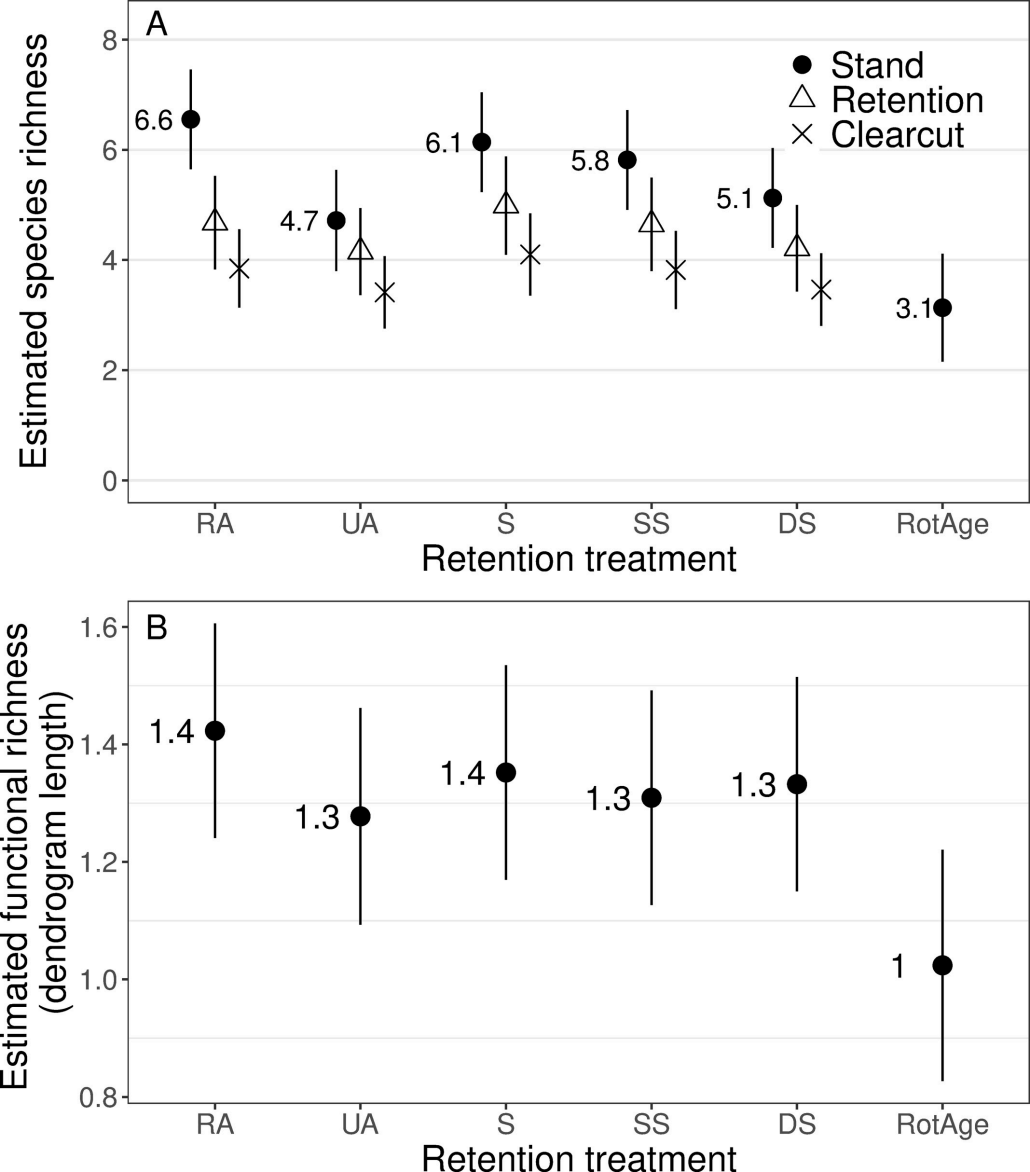
(a) Species richness estimates at the stand-scale (black circles), within retention (white triangles) and within clearcuts (Xs), and (b) stand-scale functional richness estimates, for each structural retention treatment, northwest Oregon and southwest Washington, USA, 2017–2019. Estimates represent unconditional predictions from mixed-effects models for 2018. RA = Riparian Aggregated, UA = Upland Aggregated, S = Split, SS, Split with Snags, and DS = Dispersed with Snags.

We did not find statistical support for year by treatment interactions on species or functional richness (S1 Table), so we based inference on the treatment main effects model. Furthermore, we found no evidence for an effect of temporal timing of sampling on stand-scale species richness (β = -0.01, 95% CI = -0.09–0.07) or functional richness (β = -0.07, 95% CI = -0.11–0.04), thus did not retain this variable in final models. Coefficient estimates for the effect of retention treatment on species richness at the stand scale were negative relative to RA (Table 1), but effect sizes were small. Species richness for the two treatments with only upland retention (UA and DS) were significantly lower than RA (Table 1). On average, UA and DS treatments had 1.8 and 1.4 fewer small mammal species, respectively, than the RA treatment (Fig 4A). Median species richness across all sites was low (5), and loss of 1–2 species in the UA treatment represented a ~25% reduction in species richness. Rotation-aged, unharvested forest also had significantly lower species richness relative to RA (Table 1), but sampling effort in this treatment was half the effort of other treatments. Functional richness did not differ among treatments, however functional richness was also significantly lower in rotation-aged forests (Fig 4B). We did not find a significant difference in species richness within each cover type (i.e., retention patch and clearcut) among treatments (Table 1), indicating the higher stand scale species richness in the RA treatment likely resulted from less redundancy in species composition between patches and clearcuts, not more species in the RA patches compared to patches in other treatments. Aside from lower ground cover of ferns in the dispersed treatment we did not find significant differences in coarse measures of ground vegetation among treatments (Table 2). Shrub cover was significantly higher in patches than clearcuts (Table 2).

**Table 1:** Coefficient estimates and 95% confidence intervals from linear mixed-effects models (Gaussian response) predicting stand-scale small mammal species and functional richness by structural retention treatment, northwest Oregon and southwest Washington, USA, 2017–2019.

Model			
Treatment	Stand Species Richness	Stand Functional Richness	Cover type Species Richness
Upland Aggregate (UA)	**-1.84, -3.08 –-0.59**	-0.22, -0.51–0.07	-0.12, -0.38–0.14
Split (S)	-0.41, -1.64–0.82	-0.13, -0.42–0.16	0.06, -0.19–0.31
Split with Snags (SS)	-0.74, -1.96–0.50	-0.15, -0.44–0.14	0.01, -0.26–0.24
Dispersed with Snags (DS)	**-1.46, -2.66 –-0.19**	-0.18, -0.48–0.11	-0.10, -0.36–0.15
Rotation-aged	**-3.41, -4.70 –-2.13**	**-0.74, -1.04 –-0.43**	-

Also, coefficient estimates and 95% confidence intervals for a linear mixed-effects model (Poisson response) predicting small mammal species richness by cover type (retention patch or clearcut) within structural retention treatment stands. Reference treatment for all models is Riparian Aggregate (RA). Bold values indicate significant effects with 95% confidence intervals that do not overlap zero.

**Table 2:** Coefficient estimates and 95% confidence intervals from linear mixed-effects models (Gaussian response) relating percent cover of four vegetation groups to retention treatment and cover type (patch or clearcut), northwest Oregon and southwest Washington, USA, 2017–2019.

Treatment	Percent Fern	Percent Forb	Percent Grass	Percent Shrub
Upland Aggregate (UA)	**-5.8, -10.9 –-0.6**	-3.3, -9.8–3.2	3.3, -0.5–7.1	-0.4, -1.2–4.5
Split (S)	-3.5, -8.72–1.7	-3.2, -9.7–3.3	0.8, -3.0–4.5	0.3, -0.5–1.1
Split, Created Snags	-6.6, -2.1–5.5	3.7, -2.8–10.3	1.7, -2.1–5.5	-0.50, -1.3–0.3
Dispersed (DS)	**-11.0, -16.3 –-5.8**	-5.1, -11.0–1.4	3.0, -0.7–6.8	0.5, -0.3–1.3
Cover Type	0.18, -3.1–3.5	**-6.8, -11.0 –-2.7**	-1.9, -4.3–0.5	**3.8, 3.3–4.3**

Shrub cover was square root transformed. Reference treatment for all models is Riparian Aggregate (RA). Bold values indicate significant effects with 95% confidence intervals that do not overlap zero.

**Fig 5:**
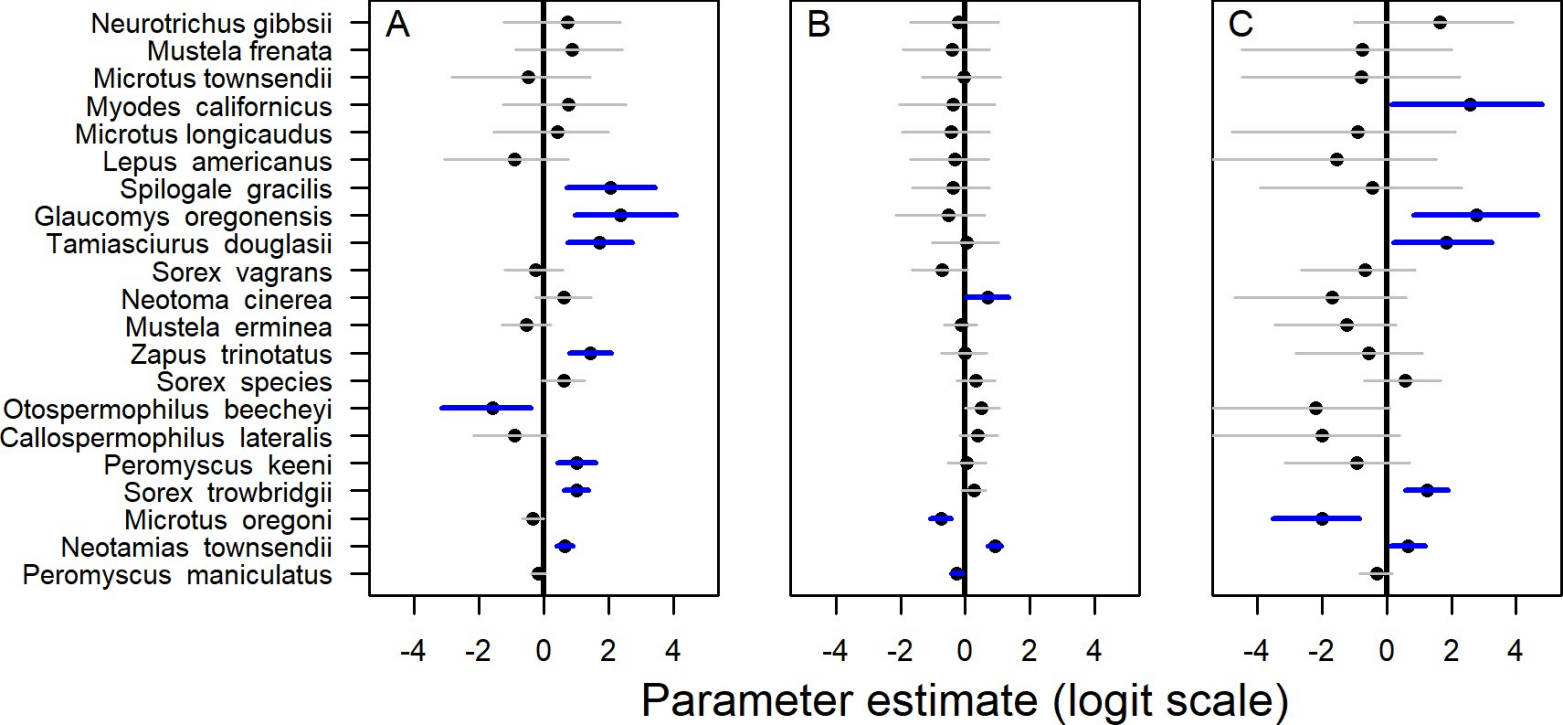
Coefficient estimates (points) and 95% Bayesian credible intervals (horizontal lines) for effects of three subplot types, (a) riparian patches, (b) upland patches, and (c) rotation-aged forests, on occupancy of 21 small mammal species compared to clearcut subplots. 95% credible intervals that do not include zero (black line) are indicated in blue. Estimates are from a community occupancy model with a sample size of 59 stands and 209 subplots within stands, sampled over three years, 2017–2019, northwest Oregon and southwest Washington, USA.

Results from the community occupancy model indicated that occupancy was significantly higher for 7 species in riparian patches and significantly lower for only one species relative to harvested areas (Fig 5A). Species with significantly higher occupancy in riparian patches included both arboreal species, each of which had higher occupancy than rotation-aged forests (Fig 5C). In contrast, occupancy of only two species was significantly higher in upland retention compared to clearcuts (Fig 5B), including an uncommon species the bushy-tailed woodrat (*Neotoma cinerea*). The occupancy of two common species was significantly lower in upland patches compared to harvested areas (Fig 5B).

## Discussion

Using a manipulative experiment, we investigated how the pattern and location of unharvested forest patches influenced small mammal community diversity. Species richness typically increases with environmental heterogeneity [13] and at a constant amount of habitat, more small patches often support higher species richness than fewer large patches [12, 44, 45]. In contrast, we found that both the dispersed treatment with multiple patches and aggregated upland treatment with one patch had low species richness compared to riparian aggregated patches. This finding indicates that rather than patch size and number, the location of patches, in this case whether upland or riparian, may be more consequential for community species richness. Our findings may contrast with results from other studies because the small (~15 tree) dispersed patches we studied were below a potential threshold size required to provide sufficient habitat for additional species not present in clearcut forests. Furthermore, additional forest habitat provided by riparian buffers likely contributed to the higher species richness observed in riparian patches compared to upland patches, consistent with connectivity to adjacent forest having a strong effect on community composition within retention areas [17]. Because aggregated patches not connected to riparian corridors had the lowest species richness, our primary prediction that aggregated retention would increase small mammal diversity compared to more dispersed retention patterns was not supported. However, our secondary prediction that connectivity to riparian zones will increase species richness was supported.

Environmental heterogeneity is an important factor [13] driving species richness patterns, with increased heterogeneity providing more resources [46] or stabilizing predator-prey and competitive interactions between species [47]. The species-area relationship predicts that sampling a larger area increases the likelihood of encountering more species [48], likely because more heterogeneity in habitat is intersected as sampling area increases [49]. Despite likely sampling a larger area in the dispersed treatment, multiple patches across this treatment had lower total species richness than the riparian-aggregated treatment. This pattern suggests that the environment across upland portions of clearcuts is relatively homogenous and only riparian zones create sufficient levels of habitat heterogeneity to increase small mammal species richness.

Data collected on vegetation in treatment stands provides some support for this conclusion. We did not observe many consistent changes in coarse measures of vegetation composition among treatments, with the exception of lower fern cover in the dispersed treatment and to a lesser degree in the upland aggregated treatment compared to treatments with riparian retention (Table 2). Ferns do not directly provide important resources for small mammals but this difference in vegetation composition between riparian and upland patches suggests that riparian retention supports different environmental conditions compared to clearcut upland areas. This heterogeneity created by riparian areas, in turn increases stand-scale small mammal species richness. However, increased small mammal richness in riparian patches could also be related to differences in species composition of vegetation in riparian zones, for example more berry producing shrubs, that our coarse measures of vegetation composition did not capture.

In addition to unique characteristics of riparian zones, the connectivity of riparian patches to additional forested habitat in riparian zones likely contributed to higher species richness between these areas and upland patches. Forest small mammal occurrence is sensitive to the amount of forest in the landscape surrounding sites [50, 51] and the movement of arboreal small mammals such as flying squirrels (*Glaucomys spp*.) is influenced by forest connectivity in managed forest landscapes [52]. We found higher occupancy of the arboreal species in the community, Humboldt’s flying squirrel (*Glaucomys oregonensis)* and Douglas’ squirrel (*Tamiasciurus hudsonius)*, in riparian patches and except for rotation-aged stands, we never encountered flying squirrels outside of riparian patches. Hence, higher species richness in the riparian-aggregated treatment is likely related to the heterogeneity and connectivity provided by forested riparian buffers. These findings build on previous research demonstrating the importance of forested riparian buffers for conserving small mammal diversity in harvested forests [53, 54]. As shown with other taxonomic groups [55], connecting retention trees with these forested corridors may be the most effective way to implement retention to conserve unique species not found in clearcut forests.

In contrast to species richness in our study, functional richness (quantified by body size, broad diet guilds, and activity strata) did not vary consistently by treatment, suggesting that lower species richness in aggregated upland patches was due to loss of functionally similar and not unique species [24]. Hence, while green tree retention patterns do not consistently alter the small mammal functional groups present in clearcut stands, upland aggregates likely have less functional redundancy. This is important because communities with lower functional redundancy may have lower stability and resiliency to future disturbance [56]. Although we used only limited trait information to define functional identity of species, the inclusion of more traits leads functional richness estimates to converge on species richness [57], and our dendrogram was successful at separating broad categories of species (e.g., rodents vs insectivores). Furthermore, the community occupancy model revealed that detection probabilities for many rare species, including arboreal species, were sufficient to estimate species-specific occupancy relationships, suggesting imperfect detection did not strongly bias functional richness estimates [58]. For example, daily baseline detection for flying squirrels was 0.35 (S1 Appendix), indicating that over a 4-day trapping session, we can be 85% confident we observed the true occupancy state for this species (i.e., detected them if present, S1 Appendix).

Our findings are consistent with previous studies in the Pacific Northwest that targeted smaller-bodied species, which found small mammal species richness was higher in riparian zones with only one species occurring at higher abundances in upland forests [59, 60]. The only species more common in upland forests, western red-backed vole (*Myodes californicus*), responds negatively to forest harvesting [61] and was rare in our data (4 individuals). The scarcity of this species on intensively managed forest landscapes may in part explain why upland patches did not contribute to small mammal species richness. *M*. *californicus* (and *M*. *gapperi* in WA) is a dominant component of old growth small mammal communities in the region [62] and its absence at our sites may reflect a lack of structural complexity in intensively managed forests, or isolation from source populations. Whether green tree retention rules in the Pacific Northwest can increase structural complexity in production forests to the point where late seral species benefit [63, 64] is an important question for management and research consideration. Similar to our findings, previous studies of small mammal abundance in other regions found limited differences in small mammal occurrence between large and small retention patches [17]. However, studies in deciduous forests of eastern North America found higher small mammal diversity in clearcut stands with aggregated retention than those without retention [65]. We did not include stands lacking retention, but our results suggest the benefits of aggregated retention may be conferred only when those patches are connected to riparian zones under current silvicultural practices in the Pacific Northwest.

## Conclusions

Across the range of clearcut sizes we evaluated, increases in small mammal richness were associated with retaining groups of trees connected to unharvested riparian corridors. In addition, only one species had higher occupancy in upland patches that did not also have higher occupancy in riparian patches compared to clearcuts, further emphasizing the value of riparian patches to small mammal diversity in recently harvested conifer plantations. For stands without riparian zones, grouping retention trees into a single patch or several small patches will result in similar species richness of small mammals. However, previous studies suggest that changes in abundance of common species in response to retention pattern should also be considered when making decisions on retention placement [66]. Retention pattern had minimal effect on functional richness of the small mammal community, but may influence levels of functional redundancy, and hence, resiliency of the community. In the early-seral stage, retention placement decisions are more consequential for small mammal species richness than the presence of functional groups.

## Supporting information

S1 Table Coefficient estimates and 95% confidence intervals from linear mixed effects models (Gaussian response) predicting small mammal species and functional richness by structural retention treatment with a treatment by sampling year interaction, northwest Oregon and southwest Washington, USA, 2017–2019. Reference treatment for all models is Riparian Aggregate (RA).
(DOCX)

S1 Fig Aerial photos depicting the arrangements of live-trapping grids (white crosses) within the different treatment types: (a) Upland Aggregated, (b) Riparian Aggregated, (c) Split, and (d) Dispersed with snags, northwest Oregon and southwest Washington, USA, 2017–2019. Sampling in the Split with Snags treatment was identical to sampling in the Split treatment. Minor differences in the configuration of grids placed in retention patches due to patch shape is apparent in panels (c) and (d).
(DOCX)

S2 Fig Number of individuals captured for each of 21 small mammal species captured >1 time in 50 clearcut treatment stands with retention and nine rotation-aged forests, northwest Oregon and southwest Washington, USA, 2017–2019. (DOCX)

S3 Fig Functional dendrogram for 21 small mammal species used to calculate functional richness within 50 clearcut treatment stands and nine rotation-aged forests, northwest Oregon and southwest Washington, USA, 2017–2019. Dendrogram was constructed using Gower’s distance and the UPGMA clustering algorithm. The functional traits used were body size, diet, and activity stratum.
(DOCX)

S4 Fig Effect size (dots) and 95% confidence intervals (horizontal lines) for random intercepts estimated for each experimental treatment stand (a and c) and experimental block (b and d) for species richness (a and b) and functional richness (b and d) from the stand-scale models, northwest Oregon and southwest Washington, USA, 2017–2019.
(DOCX)

S1 Appendix Code used to fit community occupancy model in JAGS to small mammal detection data and create Fig 4. Results of goodness-of-fit test and plot of percent confidence in species detection.
(DOCX)
