# Supplementary material for: Short-term responses of small mammal diversity to varying stand-scale patterns of retention tree patches
Source: PLoS One. 2022 Aug 31;17(8):e0273630. doi: 10.1371/journal.pone.0273630 (PMC9432693; doi:10.1371/journal.pone.0273630)
Supplement: S1 Table — Reference treatment for all models is Riparian Aggregate (RA). (DOCX) [file pone.0273630.s001.docx]

| Table S1. Coefficient estimates and 95% confidence intervals from linear mixed effects models (Gaussian response) predicting small mammal species and functional richness by structural retention treatment with a treatment by sampling year interaction, northwest Oregon and southwest Washington, USA, 2017-2019. Reference treatment for all models is Riparian Aggregate (RA). Bold values indicate effects with 95% confidence intervals that do not overlap zero. | | |
| --- | --- | --- |
|  | Model | |
| Treatment | Species richness | Functional richness |
| Upland Aggregate (UA)  Split (S)  Split, Created Snags (SS)  Dispersed (DS)  Rotation-aged  Year  Upland Aggregate * Year  Split * Year  Split with Snags * Year  Dispersed with Snags * Year  Rotation-aged * Year | **-2.09, -3.80 – -0.40**  -0.01, -1.72 – 1.68  -1.19, -2.89 – 0.51  -1.42, -3.11 – 0.28  **-3.34, -5.09 – -1.59**  -0.03, -0.87 – 0.81  0.27, -0.93 – 1.47  -0.47, -1.58 – 0.79  0.36, -0.73 – 1.64  0.04, -1.19 – 1.18  -0.09, -1.36 – 1.18 | -0.15, -0.55 – 0.24  0.07, -0.33 – 0.47  -0.16, -0.56 – 0.23  -0.09, -0.48 – 0.31  **-0.57, -0.98 – -0.16**  0.11, -0.08 – 0.31  -0.07, -0.35 – 0.21  -0.20, -0.48– 0.08  0.01, -0.26 – 0.29  -0.09, -0.37 – 0.18  -0.17, -0.46 – 0.12 |
