# Supplementary material for: Short-term responses of small mammal diversity to varying stand-scale patterns of retention tree patches
Source: PLoS One. 2022 Aug 31;17(8):e0273630. doi: 10.1371/journal.pone.0273630 (PMC9432693; doi:10.1371/journal.pone.0273630)
Supplement: S1 Fig — Aerial photos depicting the arrangements of live-trapping grids (white crosses) within the different treatment types: (a) Upland Aggregated, (b) Riparian Aggregated, (c) Split, and (d) Dispersed with snags, northwest Oregon and southwest Washington, USA, 2017–2019. Sampling in the Split with Snags treatment was identical to sampling in the Split treatment. Minor differences in the configuration of grids placed in retention patches due to patch shape is apparent in panels (c) and (d). (DOCX) [file pone.0273630.s002.docx]

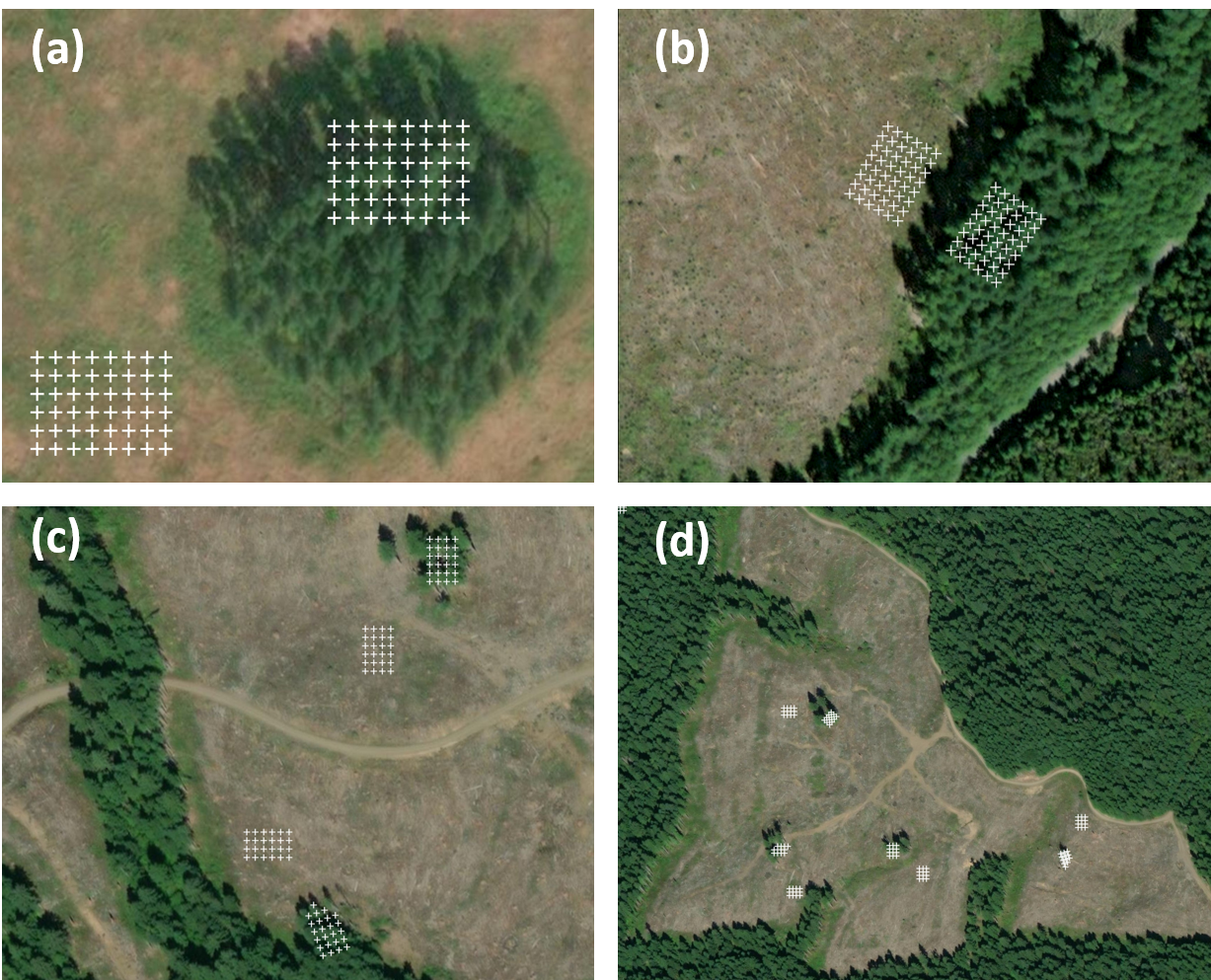


Fig S1: Aerial photos depicting the arrangements of live-trapping grids (white crosses) within the different treatment types: (a) Upland Aggregated, (b) Riparian Aggregated, (c) Split, and (d) Dispersed with snags, northwest Oregon and southwest Washington, USA, 2017-2019.. Sampling in the Split with Snags treatment was identical to sampling in the Split treatment. Minor differences in the configuration of grids placed in retention patches due to patch shape is apparent in panels (c) and (d).
