# Supplementary material for: Short-term responses of small mammal diversity to varying stand-scale patterns of retention tree patches
Source: PLoS One. 2022 Aug 31;17(8):e0273630. doi: 10.1371/journal.pone.0273630 (PMC9432693; doi:10.1371/journal.pone.0273630)
Supplement: S2 Fig — (DOCX) [file pone.0273630.s003.docx]

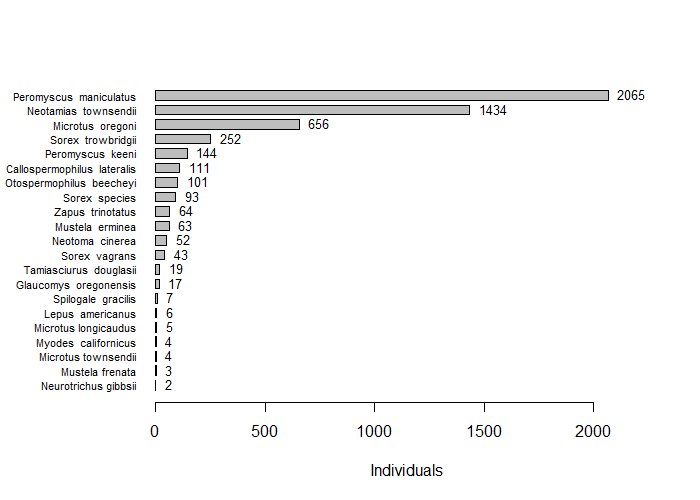


Fig S2: Number of individuals for each of 21 small mammal species captured >1 time in 50 clearcut treatment stands with retention and nine rotation-aged forests, northwest Oregon and southwest Washington, USA, 2017-2019.
