# Supplementary material for: Short-term responses of small mammal diversity to varying stand-scale patterns of retention tree patches
Source: PLoS One. 2022 Aug 31;17(8):e0273630. doi: 10.1371/journal.pone.0273630 (PMC9432693; doi:10.1371/journal.pone.0273630)
Supplement: S3 Fig — Dendrogram was constructed using Gower’s distance and the UPGMA clustering algorithm. The functional traits used were body size, diet, and activity stratum. (DOCX) [file pone.0273630.s004.docx]

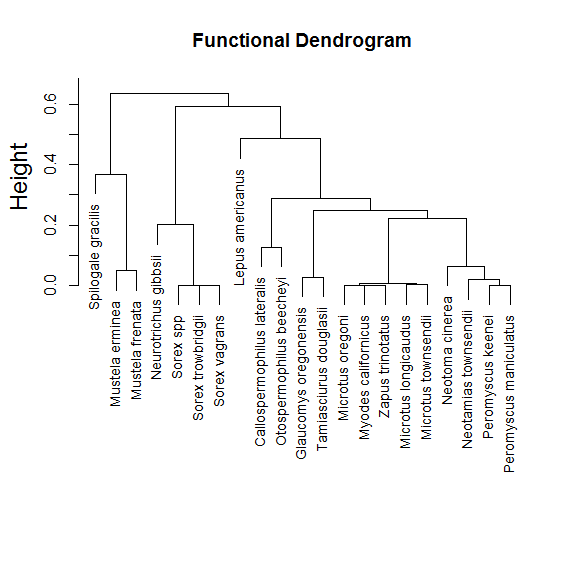


Fig S3: Functional dendrogram for 21 small mammal species used to calculate functional richness within 50 clearcut treatment stands and nine rotation-aged forests, northwest Oregon and southwest Washington, USA, 2017-2019. Dendrogram was constructed using Gower’s distance and the UPGMA clustering algorithm. The functional traits used were body size, diet, and activity stratum.
