# Supplementary material for: Short-term responses of small mammal diversity to varying stand-scale patterns of retention tree patches
Source: PLoS One. 2022 Aug 31;17(8):e0273630. doi: 10.1371/journal.pone.0273630 (PMC9432693; doi:10.1371/journal.pone.0273630)
Supplement: S4 Fig — Effect size (dots) and 95% confidence intervals (horizontal lines) for random intercepts estimated for each experimental treatment stand (a and c) and experimental block (b and d) for species richness (a and b) and functional richness (b and d) from the stand-scale models, northwest Oregon and southwest Washington, USA, 2017–2019. (DOCX) [file pone.0273630.s005.docx]

Fig S4: Effect size (dots) and 95% confidence intervals (horizontal lines) for random intercepts estimated for each experimental treatment stand (a and c) and experimental block (b and d) for species richness (a and b) and functional richness (b and d) from the stand-scale models, northwest Oregon and southwest Washington, USA, 2017-2019.

Stand

Block

Block

Stand


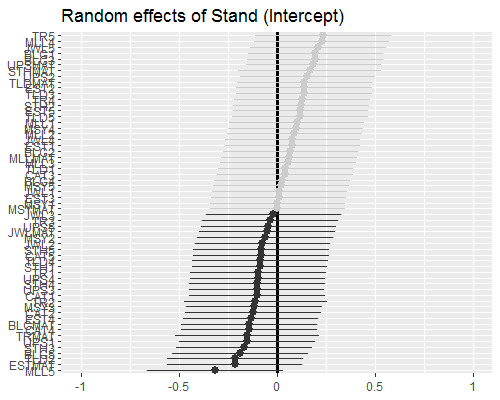

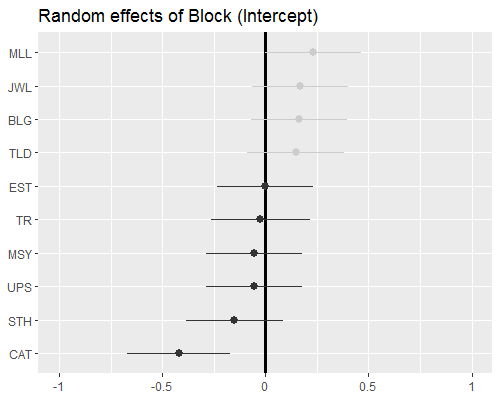

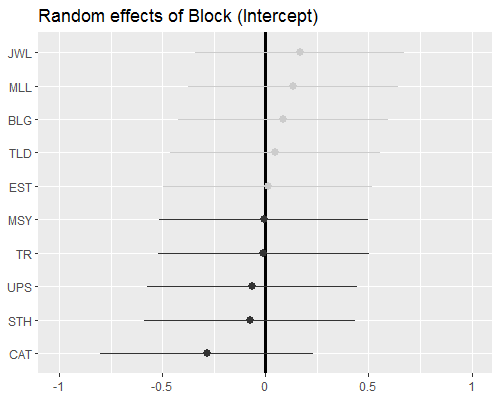

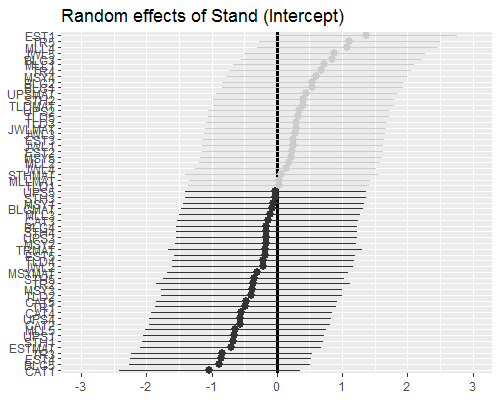


(a)

(b)

(c)

(d)
