# Supplementary material for: Short-term responses of small mammal diversity to varying stand-scale patterns of retention tree patches
Source: PLoS One. 2022 Aug 31;17(8):e0273630. doi: 10.1371/journal.pone.0273630 (PMC9432693; doi:10.1371/journal.pone.0273630)
Supplement: S1 Appendix — Results of goodness-of-fit test and plot of percent confidence in species detection. (DOCX) [file pone.0273630.s006.docx]

**S1 Appendix**

S1 Appendix. Code used to fit community occupancy model in JAGS to small mammal detection data and create Fig. 4. Results of goodness-of-fit test and plot of percent confidence in species detection.

model{

#proportion of subplots where the species occurs

psi ~ dunif(0,1)

#hyper-parameters for occupancy random species effect

mu.u ~ dnorm(0,0.001)###intercept

sigma.u ~ dunif(0,10)

tau.u <- pow(sigma.u,-2)

#hyper-parameters for detection random species effect

mu.v ~ dnorm(0,0.001)

sigma.v ~ dunif(0,10)

tau.v <- pow(sigma.v,-2)

#hyper-parameters for patch type effects

mu.beta.rip ~ dnorm(0,0.01)

tau.beta.rip <- pow(sd.beta.rip,-2)

sd.beta.rip ~ dunif(0,4)

mu.beta.up ~ dnorm(0,0.01)

tau.beta.up <- pow(sd.beta.up,-2)

sd.beta.up ~ dunif(0,4)

mu.beta.mat ~ dnorm(0,0.01)

tau.beta.mat <- pow(sd.beta.mat,-2)

sd.beta.mat ~ dunif(0,4)

#hyper-parameters for week effect on detection

mu.v.week ~ dnorm(0,0.001)

sigma.v.week ~ dunif(0,10)

tau.v.week <- pow(sigma.v.week,-2)

###random site level effects

#####site level random effect on occupancy

for(i in 1:59){

b.site[i] ~ dnorm(0, tau.site)

}

sigma.site ~ dunif(0,10)

tau.site <- pow(sigma.site, -2)

#loop over all species

for (i in 1:(n)) {

#mean for all species from the community level prior distributions

v[i] ~ dnorm(mu.v, tau.v) #species specific detection

u[i] ~ dnorm(mu.u, tau.u) #species specific abundance

beta.rip[i] ~ dnorm(mu.beta.rip,tau.beta.rip)

beta.up[i] ~ dnorm(mu.beta.up,tau.beta.up)

beta.mat[i] ~ dnorm(mu.beta.mat,tau.beta.mat)

###fixed week effect for detection

v.week[i] ~ dnorm(mu.v.week,tau.v.week)

#loop over all trapping grids

for (j in 1:J) {

#Poisson model for abundance

log(lambda[j,i]) <- u[i] + b.site[stand.factor[j]] + beta.rip[i] * PatchType[j,2] +

beta.up[i] * PatchType[j,3] +

beta.mat[i] * PatchType[j,4]

mu.a[j,i] ~ dpois(lambda[j,i])

z[j,i] <- step(a[j,i]-1) #Occupancy

#logistic model for detection

logit(r[j,i]) <- v[i] + v.week[i]*week[j]

mu.r[j,i] <- 1-pow(1-r[j,i],a[j,i])

y[j,i] ~ dbin(mu.r[j,i],4)

#Create simulated dataset to calculate the Bayesian p-value

ynew[j,i] ~ dbin(mu.r[j,i],4)

#Pearson residuals

d[j,i]<- (y[j,i] - mu.r[j,i]*4)/sqrt((mu.r[j,i]+0.000000001)*4*(1-mu.r[j,i]-0.000000001))

dnew[j,i]<- (ynew[j,i]-mu.r[j,i]*4)/sqrt((mu.r[j,i]+0.000000001)*4*(1-mu.r[j,i]-0.000000001))

d2[j,i]<- pow(d[j,i],2)

dnew2[j,i]<- pow(dnew[j,i],2)

}

}

#Calculate the discrepancy measure

p.fit<-sum(d2[1:J,1:(n)])

p.fitnew<-sum(dnew2[1:J,1:(n)])

}


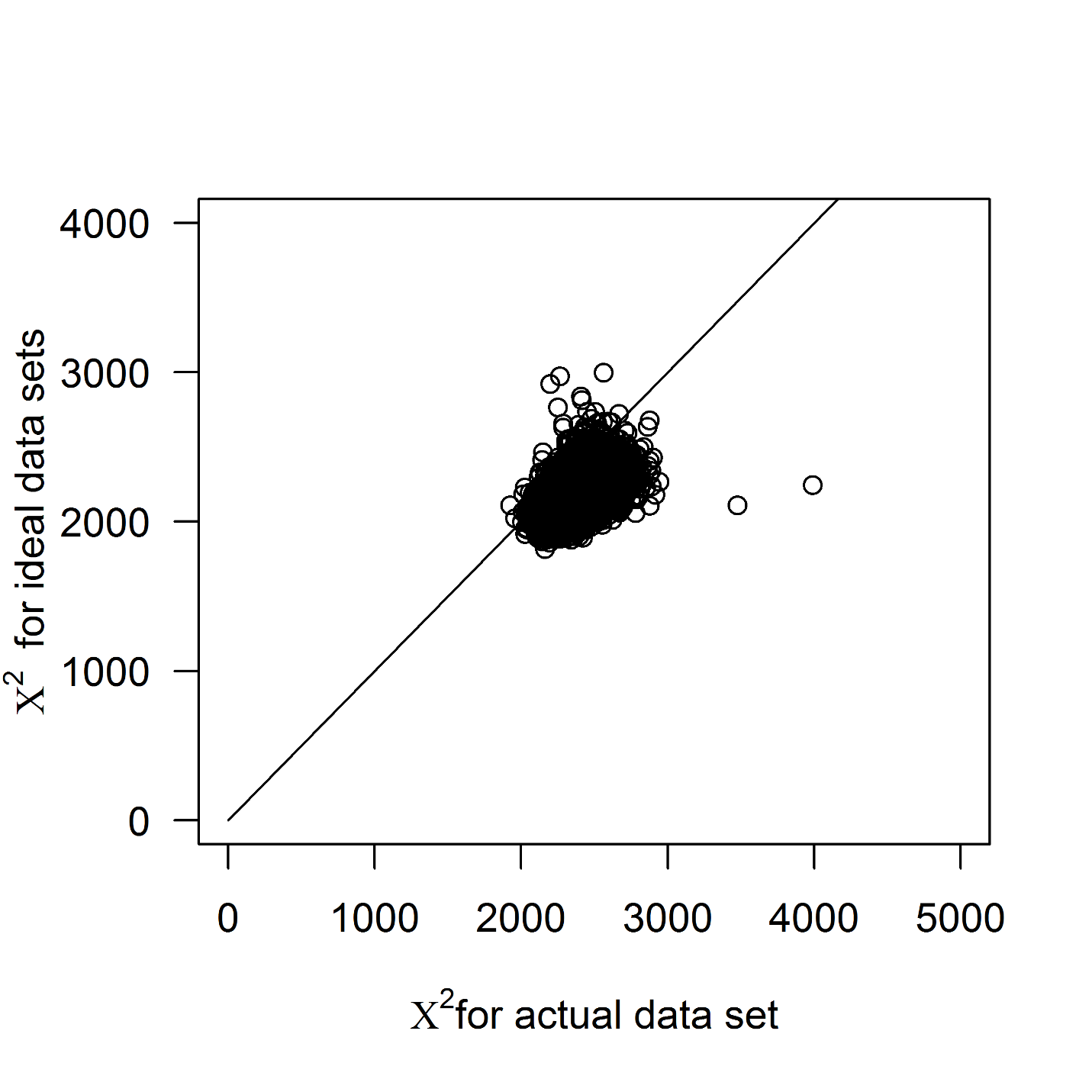


Bayesian p-value = 0.943

Result of goodness-of-fit (GOF) test using a Bayesian p-value (Gelman et al. 1996) for a Bayesian community occupancy model fit using data from northwest Oregon and southwest Washington, USA, 2017-2019. We calculated the Bayesian P-value based on the Pearson’s χ^2^ discrepancy for binomial data as Pr(χ^2^obs > χ^2^ sim) with values larger than 0.95 or smaller than 0.05 indicating a lack of fit (Tobler et al. 2015).


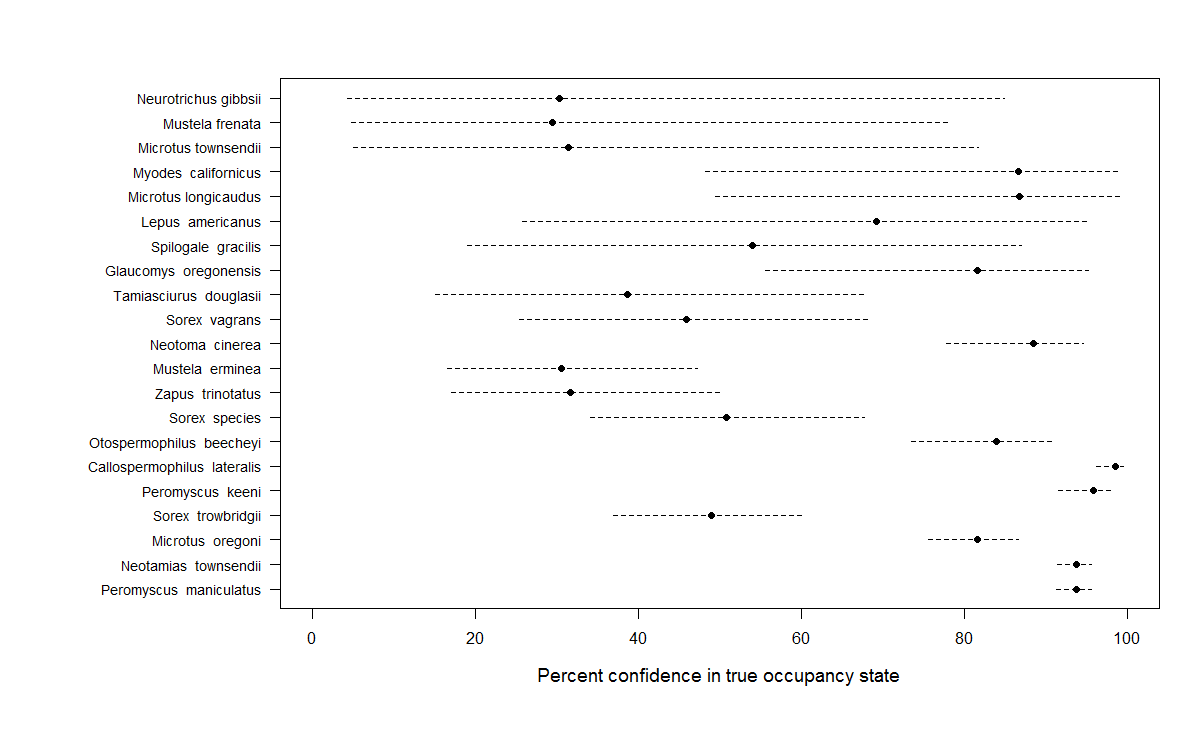


Percent confidence in the true occupancy state and 95% CI after 4 survey days for 21 species of small mammals from the community occupancy model, calculated as (1-(1-*p*)^4)*100, where *p* is species-specific baseline detection probability, using a Bayesian p-value (Gelman et al. 1996),. However, our model included heterogeneous detection probabilities caused by variable local abundance that resulted in increased detection probability, and confidence in occupancy, at sites with higher species-specific local abundance.

**Literature cited**

Gelman, A., Meng, X.-L. & Stern, H. (1996) Posterior predictive assessment of model fitness via realized discrepancies. Statistica Sinica, 6, 733-760.

Tobler, M. W., Zúñiga Hartley, A., Carrillo-Percastegui, S. E. & Powell, G. V. N. 2015 Spatiotemporal hierarchical modelling of species richness and occupancy using camera trap data. *J. Appl. Ecol.* **52**, 413–421. (doi:10.1111/1365-2664.12399)
